# Natural Antimicrobials Promote the Anti-Oxidative Inhibition of COX-2 Mediated Inflammatory Response in Primary Oral Cells Infected with *Staphylococcus aureus*, *Streptococcus pyogenes* and *Enterococcus faecalis*

**DOI:** 10.3390/antiox12051017

**Published:** 2023-04-28

**Authors:** Eugenia Butucel, Igori Balta, Iulia Adelina Bundurus, Cosmin Alin Popescu, Tiberiu Iancu, Adelina Venig, Ioan Pet, Ducu Stef, David McCleery, Lavinia Stef, Nicolae Corcionivoschi

**Affiliations:** 1 Bacteriology Branch, Veterinary Sciences Division, Agri-Food and Biosciences Institute, Belfast BT4 3SD, UK; 2 Faculty of Bioengineering of Animal Resources, University of Life Sciences King Mihai I from Timisoara, 300645 Timisoara, Romania; balta.igori@usab-tm.ro (I.B.); iulia_bundurus@animalsci-tm.ro (I.A.B.); ioanpet@usab-tm.ro (I.P.); 3 Faculty of Agriculture, University of Life Sciences King Mihai I from Timisoara, 300645 Timisoara, Romania; cosmin_popescu@usab-tm.ro; 4 Faculty of Management and Rural Tourism, University of Life Sciences King Mihai I from Timisoara, 300645 Timisoara, Romania; tiberiuiancu@usab-tm.ro; 5 Faculty of Environmental Protection, University of Oradea, 410087 Oradea, Romania; adelina.venig@uoradea.ro; 6 Faculty of Food Engineering, University of Life Sciences King Mihai I from Timisoara, 300645 Timisoara, Romania; ducustef@usab-tm.ro

**Keywords:** *Staphylococcus aureus*, *Streptococcus pyogenes*, *Enterococcus faecalis*, oral infection, natural antimicrobials, canine primary oral epithelial cells, periodontitis in dogs

## Abstract

*Staphylococcus aureus*, *Streptococcus pyogenes* and *Enterococcus faecalis* can colonize the tooth root canals, adhere to dentin walls, and frequently cause periodontitis in dogs. Bacterial periodontal diseases are common in domesticated pets, causing severe oral cavity inflammation and a strong immune response. This study investigates the antioxidant effect of a natural antimicrobial mixture (Auraguard—Ag) on the ability of *S. aureus*, *S. pyogenes* and *E. faecalis* to infect primary canine oral epithelial cells as well as its impact on their virulence factors. Our data show that a concentration of 0.25% Ag is sufficient to inhibit the growth of all three pathogens, whereas a concentration of 0.5% will become bactericidal. The sub-inhibitory concentration of 0.125% Ag reveals that the antimicrobial mixture can significantly reduce biofilm formation and exopolysaccharide production. The impact on these virulence factors was further translated into a significantly reduced ability to infect primary canine oral epithelial cells and restore epithelial tight junctions, with no impact on the epithelial cell viability. The post-infection inflammatory cytokines (IL-1β and IL-8) and the COX-2 mediator were also reduced both in mRNA and protein expression levels. The oxidative burst, detected upon infection, was also decreased in the presence of Ag, as our results show a significant decrease in H_2_O_2_ released by the infected cells. We show that inhibition of either NADPH or ERK activity will result in a downregulation of COX-2 expression and lower levels of H_2_O_2_ in infected cells. Conclusively, our study shows that natural antimicrobials reduce pro-inflammatory events, post infection, through an antioxidative mechanism that involves the downregulation of the COX-2 mediator via the inactivation of ERK in the absence of H_2_O_2_. As a result, they significantly reduce the risk of secondary bacterial infections and host oxidative stress caused by *Staphylococcus aureus*, *Streptococcus pyogenes* and *Enterococcus faecalis* accumulation in biofilms in an in vitro canine oral infection model.

## 1. Introduction

Given the high prevalence of periodontal diseases in dogs and the difficulties faced by veterinarians in treating such diseases [1], the availability of natural alternatives to antibiotics is of crucial importance [2]. Dental biofilm, or plaque, has a major role in the onset of dental caries, with the oral cavity’s moist environment and the adherent surfaces fostering plaque formation, which is difficult and expensive to remove [3,4]. Most biofilm-forming bacteria originate in the bacterial plaque formed on the tooth surface [5]. Identification of biological agents, efficient in controlling biofilm formation and consequently dental plaque [6], are of constant concern; therefore, mixtures of organic acids and their effects have been studied [7].

Pathogens of the genus *Staphylococcus* are frequently isolated from dog dental plaques, which are susceptible to natural antimicrobials, as recently reported [4]. *Staphylococcus aureus* (*S. aureus*), for example, is found on the skin, nasal and oral cavity where it can cause dental caries and periodontal disease [8]. For instance, the extracts of *Salvadora persica* exhibit a significant antibacterial effect against *S. aureus*, and are considered an efficient intervention against this pathogen [9]. Most recently, from 88 extracts of *Cassia alata* (leaves, roots and stem), 32 displayed strong antimicrobial, antioxidant and anti-inflammatory activity in *S. aureus* infections [10]. Dental plaque in pet dogs also serves as a reservoir of antimicrobial resistance genes, with humans and dogs being in close contact posing a high risk of transmission [11]. This issue was emphasized in a recent study which identified that over 60% of canine plagues harbor the *Staphylococcus* genus with a high resistance to antibiotics [3]. Specifically, the *S. aureus* isolates from the dental plaque samples of dogs exhibited an elevated resistance to tetracycline, penicillin encoding and cefazolin mainly encoded by *bla*TEM, *tet*M and *mec*A genes [3].

*Streptococcus pyogenes* (*S. pyogenes*) is also frequently isolated from dental plaque and can be responsible for causing severe throat and mouth infections in humans [12]. Animals have become a new source of *S. pyogenes*, alongside human-derived *S. pyogenes* which can indeed also colonize and infect animals [13]. *S. pyogenes* also has the potential to trigger respiratory illness in pets [14]. Nasal and oral swabs from dogs and cats carried penicillin-macrolide-resistant *S. pyogenes*, which concurrently infects humans with a wide spectrum of serious conditions ranging from oral infections such as tonsillitis, respiratory diseases such as pharyngitis and leads to severe life-threatening illnesses such as endocarditis, pneumonia and encephalitis. Children who often spend a significant amount of time interacting and expressing affection towards their pets are particularly prone to contracting these strains due to their intimate contact [14]. Molecular characterization of *S. pyogenes* derived from pets suffering from respiratory illnesses, such as dogs and cats, involves oral/nasal swabs that illustrate the presence of the *erm*B gene, which accounts for macrolide resistance and is also widely responsible for heightened erythromycin, azithromycin and clindamycin resistance [14]. Natural antimicrobials can display antimicrobial effects against *S. pyogenes* by disrupting bacterial membranes, thus having the potential to be considered alternative treatments for respiratory diseases [15]. One example is carvacrol, a plant extract which induces a bactericidal effect against *S. pyogenes* at a concentration between 0.53–1.05 mM [16].

Alongside *Staphylococcus* spp. and *Streptococcus* spp., *Enterococcus* spp. is another genus often isolated and present in the canine oral cavity and recognized for its role in developing dental plaque and periodontal disease [17]. *Enterococcus faecalis* (*E. faecalis*), specifically, forms biofilms in oral cavities, contributes to oral diseases and can efficiently be reduced by natural antimicrobials [18]. The bacteriostatic effect of natural antimicrobials against *E. faecalis* on dental plaque was directly confirmed with many flavonoids (luteolin, morin, naringin, quercetin and rutin) [19]. Controlling the spread of this pathogen is of clinical importance because *E. faecalis* has the potential to enter the bloodstream and become implicated in systemic infections related to periodontal disease, a prevalent inflammatory condition in both humans and dogs resulting from a polymicrobial biofilm formed on tooth surfaces [20,21]. This bacterium exhibits various virulence factors, such as biofilm formation, expression of survival genes, modulation of the host immune system and the capacity to form mixed plaque and biofilm with other bacteria, which contributes to the enhancement of its resistance to antimicrobials [22]. Research has shown that *E. faecalis* is naturally resilient to cephalosporins and aminoglycosides and might easily acquire resistance to vancomycin and other antibiotics [22]. Similarly to *S. aureus* and *S. pyogenes*, due to frequent and intimate interactions with humans, dogs may serve as a substantial reservoir for *E. faecalis* in humans; a risk which is enhanced by the capacity of this bacterium to acquire and disseminate antibiotic resistance genes [23].

Many traditional therapeutic methods have shown to be insufficient or unsuccessful against plaque biofilms. To mitigate the risks of oral infections caused by *S. aureus*, *S. pyogenes* and *E. faecalis*, research should identify novel solutions, including new antimicrobial agents, develop alternative therapeutic strategies to antibiotics, promote effective oral hygiene practices and enhance our understanding of the complex interplay between the host and microbial factors. The aim of our study was to reveal further details of the biological mechanisms and establish the benchmark for their mechanism of action against pathogenic bacteria of plaque origin in canines. In our in vitro studies, we use primary canine oral epithelial cells to establish their antagonistic effect against *S. aureus*, *S. pyogenes* and *E. faecalis*, pathogens frequently isolated from canine dental plaque. The approach taken, using antimicrobials in a mixture, proved to be a very efficient as they seem to have increased efficiency when used in combinations [24]. This blended approach was proven to be efficient against bacteria [25], parasites [26,27] and viruses [28] both in vitro and in vivo, clearly showing their efficiency in improving gut health in a variety of hosts.

## 2. Materials and Methods

### 2.1. Bacterial Strains and Cell Lines

*Staphylococcus aureus* (*S. aureus* DSM1104)—clinical isolate (laboratory stock), *Streptococcus pyogenes* (*S. pyogenes*)—clinical isolate, and *Enterococcus faecalis* (*E. faecalis* 8260)—clinical isolate, were grown in TSAYE (Tryptone Soya Yeast Extract) at 37 °C. The D6234 oral primary epithelial cells were grown in DMEM (Cell Biologics, Chicago, IL, USA) with 10% FBS (fetal bovine serum in. 75-cm^2^ tissue culture flasks (Sigma-Aldrich, Arklow, Ireland, SIAL0641). The humidity and atmosphere were maintained at 37 °C and 5% CO_2_, respectively. Auraguard (Ag) included: 5% maltodextrin, 1% sodium chloride, 42% citric acid, 18% sodium citrate, 10% silica, 12% malic acid, 9% citrus extract and 3% olive extract (*w*/*w*). Bioscience Nutrition, Fedamore, Ireland supplied the raw materials.

### 2.2. Minimum Inhibitory (MIC) and Bactericidal Concentration (MBC)

The MIC and the MBC of Auraguard against *S. aureus*, *S. pyogenes* and *E. faecalis* strains was determined using the two-fold tube dilution method. Auraguard dilutions (8% to 0.015625% *v*/*v*) were performed in TSAYE (Tryptone Soya Yeast Extract Broth). Overnight cultures were collected via centrifugation, washed in PBS two times and re-diluted in TSAYE broth to 1  ×  10^6^ CFU/mL. Each vial was inoculated with 5 × 10^5^ CFU/mL of each bacterium. Separate bijou (5 mL) with growth media, with or without Auraguard or bacteria, were used as positive controls following growth at 37 °C for 24 h in aerobic conditions. The absence of visible growth was considered to be above the MIC. A volume of 100 mL was taken from each vial, for inoculation, and placed for 24 h at 37 °C on TSAYE agar plates. Negative controls, including TSAYE, with or without Auraguard or bacteria, were also included. The sub-inhibitory concentrations were estimated by exposing the pathogens to different concentrations of the antimicrobial mixture. All experiments were performed in triplicate and on three different occasions.

### 2.3. Biofilm Assay and Exopolysaccharide (EPS) Measurement

Bacterial adherence was investigated in a 96-well plate in order to estimate the impact on biofilm formation. The *S. aureus*, *S. pyogenes* and *E. faecalis* strains were tested. The isolates were grown in a TSAYE broth, under static conditions, grown at 37 °C for 24 h and altered to 1.5 × 10^8^. Concentrations of 0.06% and 0.125% Auraguard were used for testing as it was previously indicated as inhibitory. For this, 230 μL of each bacterial culture was added to the wells of the 96-well plate, and Auraguard was added to test its efficiency against each pathogen, in triplicate. A positive control was constructed for each isolate and included 230 μL of bacterial culture and 70 μL of sterile TSAYE broth. The negative included only 300 μL of sterile TSAYE broth. After incubation at 37 °C for 24 h, the supernatant was removed, and the pellet washed (3×) with distilled water to take the non-adhered bacteria. A control including the medium only and Auraguard was also included. Methanol (250 μL) methanol was used in each well, for 15 min, discarded, and then the plate was dried for 2 min. A volume of 250 μL of 1% crystal violet per well was added for 10 min prior to removal with tap water followed by 250 μL of 33% glacial acetic acid. Optical density in each well was determined via spectrophotometry. The data were analyzed, and the graph was designed with Prism 5 (GraphPad Software Inc., La Jolla, CA, USA). All experiments were performed in triplicate. EPS measurements were performed as previously described [29]. EPS measurements were performed as previously described with small modifications [30,31]. Bacterial cultures grown (50 mL) with or without 0.125% Auraguard were harvested via centrifugation (1500× *g* for 20 min), and the supernatant was filtered through a 0.22-µm filter. Chilled 100% ethanol was added to the filtered supernatant and stored overnight at 2 °C for EPS precipitation. Precipitated EPS was collected via centrifugation at 10,000× *g* for 20 min. Ethanol precipitation was repeated three times. To eliminate low-molecular-weight polysaccharides, the resulting pellet was dissolved in distilled H_2_O_2_ and dialyzed for 48 h at 2 °C by using dialysis tubes (2000–3500 Da cutoff). The resulting dialysate was frozen at −20 °C for future use. EPS was quantified using the colorimetric phenol-sulfuric acid method as previously described [32].

### 2.4. Infection Assay

The D6234 oral primary epithelial cells were grown in DMEM (Cell Biologics, USA) in the presence of 10% FBS. The cells were maintained in 75-cm^2^ tissue culture flasks (Sigma-Aldrich, Arklow, Ireland, SIAL0641) at 37 °C with 5% CO_2_ under a controlled humidity. To test the effect of Auraguard on the ability of *S. aureus*, *S. pyogenes* and *E. faecalis* to adhere to and invade D6234, the assay was performed as previously described [33]. The D6234 cells (5.5 × 10^5^ cells per well) were cultured for 22–24 h to reach 80–90% confluence in six-well tissue culture plates. The pH was constantly at neutral values (pH 7.2). Cultures of *S. aureus*, *S. pyogenes* and *E. faecalis* were suspended in a DMEM medium at OD_600_ of ≈ 0.3. Cells were washed (2×) in a DMEM medium with 10% FBS. A volume of 2 mL of the DMEM medium was added to each well followed by a concentration of 0.125% Auraguard and bacteria were used to reach an MOI (multiplicity of infection) of 100. The plates were gently centrifuged at 250× *g* for 5 min and incubated for a further 3 h. The cell-associated bacteria were estimated by washing the infected monolayers (3×) with PBS followed by exposure to 0.1% Triton X-100 in PBS for 15 min at 41.5 °C and 37 °C. Dilutions (10-fold) of infected or control wells were plated onto TSAYE agar and incubated for 2 days prior to enumeration, at 37 °C. All assays were performed in triplicate and on three separate days. Cytotoxicity of Auraguard was determined as previously described [34] by utilizing the MTT assay (Sigma-Aldrich, Gillingham, England, UK).

### 2.5. Transepithelial Electrical Resistance (TEER)

The D6234 oral primary epithelial cells were seeded in transwells (5  ×  10^4^; 6.5-mm diameter; 0.4-μm pore size; Corning) and allowed to form apical junctional complexes. Seeded and infected transwells, with either *S. aureus*, *S. pyogenes* or *E. faecalis*, were used to measure TEER after 3 h using an EVOM X meter connected to an Endohm chamber (World Precision Instruments). All experiments were performed at least three times. The mean (SD) was calculated for each assay.

### 2.6. Gene Expression and Protein Lysate Analysis

The quantification of IL-1β, COX-2 and IL-8 gene expression was conducted as previously described with little changes [35]. Briefly, the infected D6234 cells were frozen in liquid nitrogen until use. The RNeasy Plus Mini Kit (Qiagen, Manchester, UK) kit was used for RNA isolation. Reverse-transcribed RNA was obtained by using the Transcriptor First Strand cDNA Synthesis Kit (Roche, Dublin, Ireland). Further, to generate mRNA, we performed quantitative RT-PCR using QuantiNova SYBR Green PCR Kit (Qiagen, Manchester, UK) on a LightCycler 96 (Roche). The primers used GAPDH (F:gtcttcaccaccatggagaag, R:gttgtcatggatgaccttggc), IL-1β (F:ctttgaagctgatggccctaaa, R:agtggtggtcggagattcgt), COX-2 (F: cacaggcttccattgaccaga, R: gtgctccaacttctaccatgg) and IL-8 (F: cttggcagccttcctgatttc, R: ccagacagagctctcttccat) [36]. The gene expression was normalized to GAPDH (glyceraldehyde-3-phosphate dehydrogenase). The 2^−ΔΔCT^ procedure was used to analyze the fold changes in relative expression relative to the control. The quantification of IL-1β, COX-2 and IL-8 in cell lysate was analyzed as previously described [36].

### 2.7. Measurement of Bacterial Released LDH from D6234 and Bacterial Cells

Lactate dehydrogenase release (LDH) was measured as previously described [37] using the cytotoxicity measurement kit (Roche, Buckinghamshire, UK) following the manufacturer’s guidelines. The LDH activity in the cell supernatant was investigated via the pre-treatment of D6234 cells with 0.125% and 0.25% Auraguard for 3 h. After 3 h, the supernatant was collected for LDH measurement. For bacterial measurements, after 3 h of growth, in the presence of 0.125% and 0.25% Auraguard and DMSO, *S. aureus*, *S. pyogenes* and *E. faecalis* supernatants were separated via centrifugation. All supernatants were further processed as per the manufacturer’s guidelines. Cytotoxicity was calculated as a percentage of the total cells lysed in Triton × 100.

### 2.8. Extracellular Hydrogen Peroxide (H_2_O_2_), Superoxide Dismutase (SOD) and Catalase (CAT) in Infected D6234 Cells

Hydrogen peroxide release was measured using the Amplex^®^ UltraRed/HRP (Thermo Fischer, Scientific, Horsham, UK) as previously described [35]. Briefly, the culture media (50 mL) were mixed with the Amplex^®^ UltraRed/HRP (Thermo Fischer Scientific, UK). After the addition of horseradish peroxidase, fluorescence was determined at 530-nm excitation and 590-nm emission using a fluorescence microplate reader (FLUOstar Omega, BMG Labtech). The SOD activity was established using an SOD colorimetric activity kit (Thermo Fisher, UK). Similarly, the catalase activity was established by using a catalase activity kit (Abcam, Trumpington, UK, ab83464) as per the manufacturer’s instructions. The NADPH inhibitor diphenyleneiodonium chloride (DPI, Sigma; 15 µM, 45 min preincubation and discard) was used during the 24-h measuring interval. Approximately 60 min prior to infection, a volume of 30 µM of PD98059 (Sigma-Aldrich, Gillingham, UK) was added to investigate the role of the ERK signal transduction pathway. Average values were used to obtain the mean relative fluorescence intensity. All experiments were performed in triplicate.

### 2.9. Statistical Analysis

The GraphPad Prism software (Dotmatics) and Student’s *t*-test were used to calculate the statistical significances. The significance was assigned at *p*-values < 0.05. As indicated for all methodologies, the experiments were conducted on at least three separate occasions in triplicate.

## 3. Results

### 3.1. Minimum Inhibitory Concentrations and Minimum Bactericidal Concentrations

Our first aim was to establish the MIC and the MBC concentrations of the natural antimicrobial mixture (Auraguard) for *S. aureus*, *S. pyogenes* and *E. faecalis*. Antimicrobial activity was detected at 0.25% (MIC) for all strains studied, and a minimum bactericidal concentration at 0.50% (MBC) (Table 1). Based on the MIC values detected, the concentration of 0.125% was selected to further assess their repressive effect against the infection of D6234 cells and to prevent any effect on bacterial growth.

### 3.2. Bacterial Growth, Biofilm Formation and EPS Production

**Figure 1:**
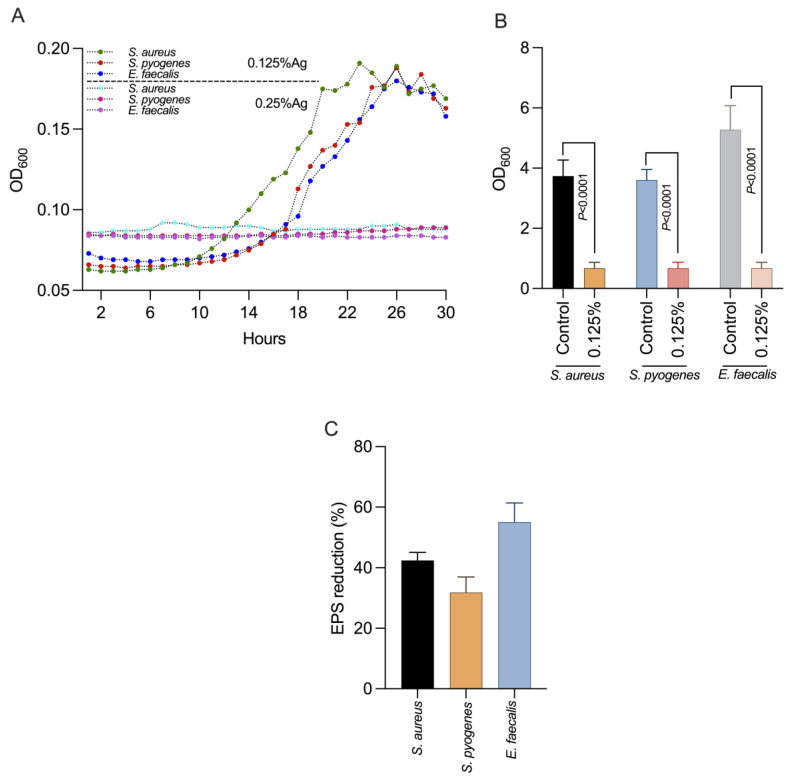
The impact on bacterial growth (**A**), biofilm (**B**) and production in EPS (**C**) of *S. aureus, S. pyogenes* and *E. faecalis* using 0.125% Auraguard. Error bars signify the standard deviation of the means of three different experiments. EPS values are shown as percentages of the control.

We next investigated the impact of 0.125% Auraguard on *S. aureus*, *S. pyogenes* and *E. faecalis* virulence factors including the formation of biofilms and EPS production. First, we showed (Figure 1A) that at the chosen concentration of 0.125% Auraguard, the antimicrobial mixture had a sub inhibitory effect and did not affect the growth of *S. aureus*, *S. pyogenes* or *E. faecalis*, allowing us to assess its biological effects. Figure 1B indicates that at a concentration of 0.125% Auraguard, the ability of *S. aureus*, *S. pyogenes* and *E. faecalis* to form a biofilm (*p* < 0.0001) was significantly reduced. The EPS measurement indicated that EPS decreased when the antimicrobial was applied (Figure 1C). A 42% reduction in *S. aureus* EPS, 30% for *S. pyogenes* and 58% for *E. faecalis* EPS was achieved. These results clearly show that Auraguard reduces the ability of *S. aureus*, *S. pyogenes* and *E. faecalis* to display some of the most significant virulence factors (biofilm and EPS), potentially leading to reduced virulence towards epithelial cells.

### 3.3. The Impact of the Natural Antimicrobial Mixture on Bacterial Membrane Integrity

**Figure 2:**
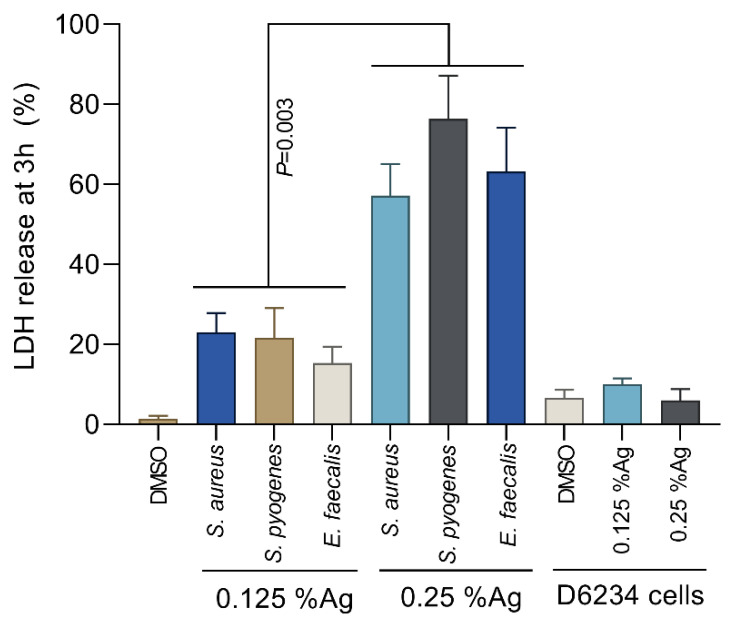
Auraguard effect on *S. aureus*, *S. pyogenes* and *E. faecalis* membrane integrity at concentrations of 0.125% and 0.25%. Data are presented as % LDH release. Error bars represent the standard deviations of three different experiments.

To assess the bacterial membrane integrity, we have performed the L-lactate dehydrogenase (LDH) membrane leakage assays following the exposure of *S. aureus*, *S. pyogenes* and *E. faecalis* to 0.125% and 0.25% Auraguard. The LDH release by *S. aureus*, *S. pyogenes* and *E. faecalis* treated with 0.125% Auraguard was significantly lower (*p* = 0.003) compared to the LDH release in the presence of 0.25% Auraguard (Figure 2). The amount of LDH released in the presence of 0.25% Auraguard confirms the results presented in Table 1, which indicates that at concentrations above 0.25%, Auraguard becomes bactericidal. Auraguard does not cause a release of LDH from exposed D6234 cells, which further confirms the non-cytotoxic effect.

### 3.4. Assessment of Auraguard Impact D6234 Infection Levels, TEER and Cell Cytotoxicity

**Figure 3:**
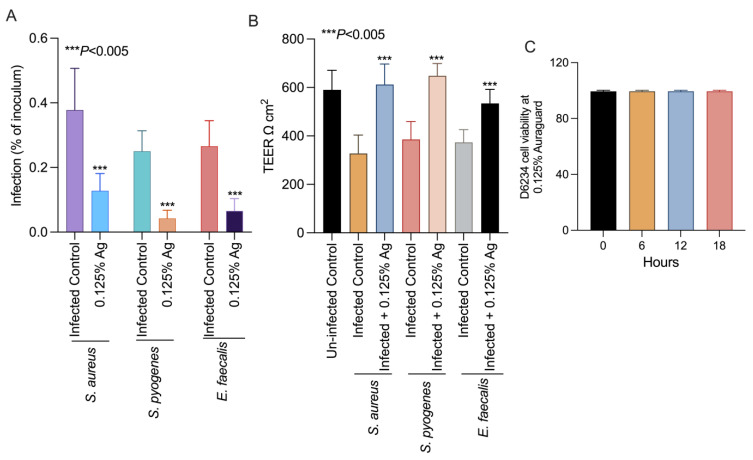
In vitro effect of Auraguard on the adherence of *S. aureus*, *S. pyogenes* and *E. faecalis* to pre-treated D6234 cells (**A**), TEER (**B**) and cell viability in the presence of 0.125% Ag (**C**). The adherence results are expressed as the percentages of the initial inoculum. Error bars represent the standard deviations of three different experiments. The IL-1 β, COX-2 and IL-8 expressions in *S. aureus*-, *S. pyogenes*- and *E. faecalis*-infected D6234 cells.

To further prove that the attenuation of bacterial virulence factors could also impact on their virulence, we set up an in vitro infection assay to test the ability of *S. aureus, S. pyogenes* and *E. faecalis* to infect the D6234 primary canine oral epithelial cell line. Incubation of the D6234 with 0.125% Auraguard during infection significantly reduced the ability of all three pathogens (*p* < 0.005) to adhere to the D6234 cells (Figure 3A) after 3 h of infection. Moreover, the reduction in total adherence was accompanied by increased TEER when infected D6234 cells were exposed to 0.125% Auraguard (Figure 3B). Figure 3C indicates that at the concentration of 0.125% Auraguard, the viability of the D6234 cells was not affected. These results suggest that 0.125% Auraguard is capable of preventing the adherence of *S. aureus*, *S. pyogenes* and *E. faecalis* to D6234 primary oral epithelial cells and to restore the cellular structures damaged upon infection.

**Figure 4:**
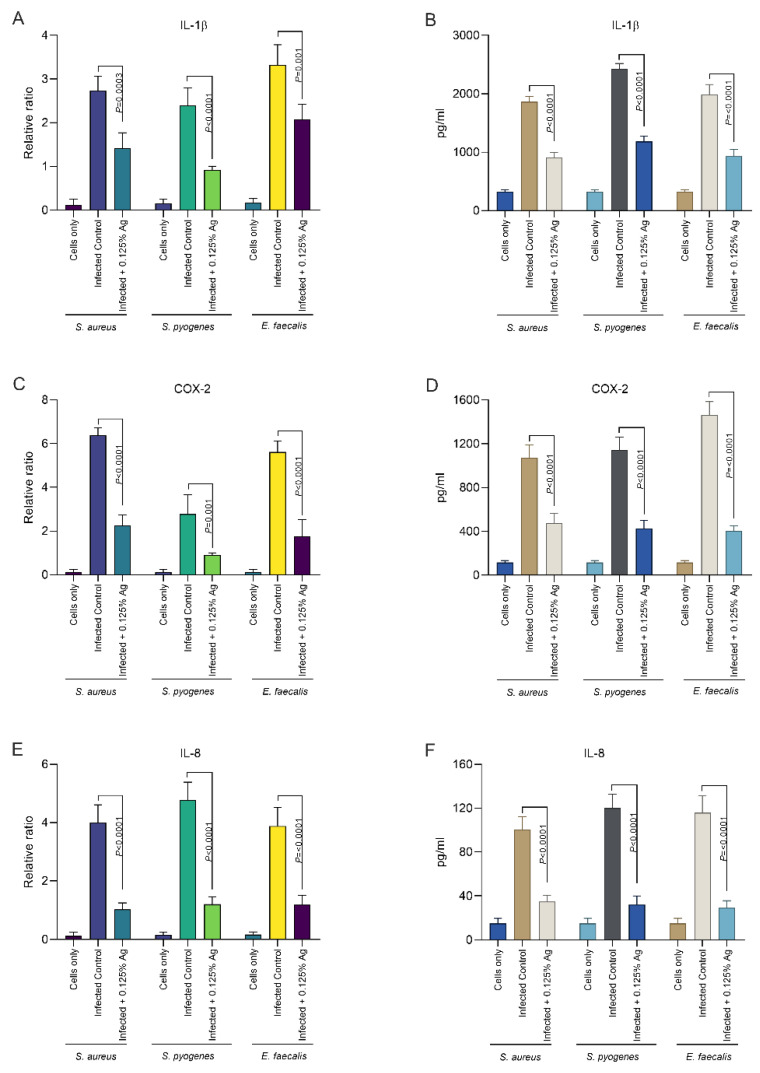
The IL-1β, COX-2 and IL-8 mRNA (**A**,**C**,**E**) and protein expression (**B**,**D**,**F**) levels in infected D6234 cells in the presence of 0.125% Auraguard. Error bars represent the standard deviations of three different experiments. A *p* value below 0.05 was considered significant and is specified on the graphs to reflect the effect of Auraguard.

Our next aim was to investigate the impact of Auraguard on the host D6234 primary canine oral epithelial cells’ inflammatory response. We have assessed if the *S. aureus*, *S. pyogenes* and *E. faecalis* induced inflammatory response can be attenuated by the natural antimicrobial mixture. The relative mRNA expression levels of IL-1β and IL-8 were measured after 3 h of infection and in the presence of 0.125% Auraguard. First, as indicated in Figure 4, both the mRNA expression levels (Figure 4A) and enzyme production (Figure 4B) of IL-1β were significantly reduced in the presence of the antimicrobial mixture for all three pathogens upon infection. This effect was replicated in the case of IL-8 measurements where both the mRNA (Figure 4E) and enzyme levels (Figure 4F) were also significantly decreased. Furthermore, our data indicate that COX-2, the expression mediator of IL-1β and IL-8, was also significantly reduced, both in mRNA expression (Figure 4C) and protein levels (Figure 4D). Our data demonstrate that Auraguard has an anti-inflammatory effect, as indicated by the cytokine levels detected in infected and Auraguard treated D6234 cells. Moreover, we believe that the reduced IL-1β and IL-8 levels reflect the lower COX-2 expression levels.

### 3.5. The Impact of the Antimicrobial Mixture as an Antioxidant against Hydrogen Peroxide Formation, CAT, SOD and COX-2 Production in Infected D6234 Cells

**Figure 5:**
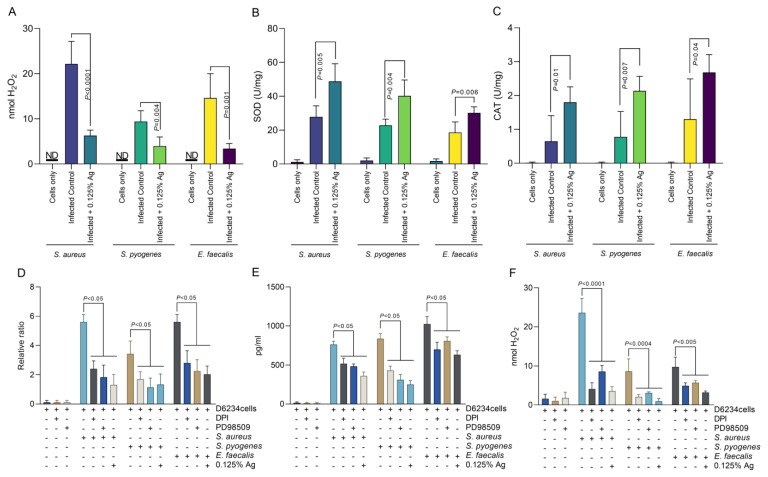
The effect of Auraguard on the extracellular levels of H_2_O_2_ released by *S. aureus*-, *S. pyogenes*- and *E. faecalis*-infected D6234 cells. (**A**,**B**) SOD activity, (**C**) CAT activity. (**D**) indicates the COX-2 relative gene expression, and (**E**) shows COX-2 protein production in the presence of DPI and PD98509 inhibitors. The H_2_O_2_ production during DPI and PD985509 inhibition is shown in (**F**). A *p* value below 0.05 was considered significant and is indicated on the graphs to reflect the effect of Auraguard.

Based on the above results, indicating that Auraguard was involved in D6234 cell membrane restoration and reduced inflammatory effects, we have next investigated the impact on H_2_O_2_ release by the infected cells. As we indicate in Figure 5A, the presence of 0.125% Auraguard reduced the level of H_2_O_2_ released from ≈ 22 nmol to ≈ 6 nmol (*p* < 0.0001) in cells infected with *S. aureus*. Similar reductions were observed in *S. pyogenes*-infected cells, from ≈ 8 nmol to ≈3.5 nmol (*p* = 0.004), and in *E. faecalis*-infected cells, from ≈ 14 nmol to ≈3 nmol (*p* = 0.001). The decrease in H_2_O_2_ release is potentially caused by the significant rise in SOD (Figure 5B) and CAT (Figure 5C). Furthermore, we have investigated the role of H_2_O_2_ production and ERK inhibition in COX-2 regulation. Our data show that inhibition of either NADPH activity with DPI, or the inhibition of ERK activity by PD98509, have resulted in a decrease in COX-2 production by both gene expression (Figure 5D) and protein production (Figure 5E). Our results suggest that the antioxidant effect—described in Figure 5—of the antimicrobial mixture reduces not only bacterial invasion but also the release of signaling molecules, such as H_2_O_2_, which might act as a pro-inflammatory trigger. We prove that in the absence of pathogen-induced H_2_O_2_, the pro-inflammatory regulator COX-2 is downregulated, which explains the anti-inflammatory effect described in Section 3.5.

## 4. Discussion

Periodontal disease and gum inflammation in dogs can be caused by pathogenic bacteria, with origins in the dental plaque [38]. Even though dental caries are unusual in dogs, bacteria such as *E. faecalis* is commonly present [39], and solutions to reduce the impact of dental plaque bacteria on oral health and biofilm formation is of increasing concern [17]. Herein we show that natural antimicrobials in mixtures can reduce the growth of *S. aureus*, *S. pyogenes* and *E. faecalis*, and can reduce their EPS production. Moreover, this inhibitory effect is further reflected in a reduced adherence to primary oral epithelial cells, reinforced epithelial tight junctions and the unimpacted cell viability. Similar mixtures of natural antimicrobials have been recently shown to treat and alleviate inflammatory events in a variety of hosts [7,26,28,37,40] with such therapies, based on the fact that natural products are able to eliminate multispecies oral biofilms and ultimately control bacterial-caused oral diseases [41].

Periodontal disease in dogs is associated with an increased production of IL-1B and IL-8 [42] in the presence of increased COX-2. COX-2, an enzyme involved in the two-step transition of arachidonic acid, is induced by bacterial and viral infections and it is considered that its inhibition might reduce the expression of proinflammatory cytokines [43]. Natural antimicrobials, such as flavonoids and other bioactive compounds, have been shown to inhibit COX-2 activity, affecting pathogen post-inflammatory effects, including oxidative stress [44,45]. Similarly, our data also show that the antimicrobial mixture, Auraguard, can indeed reduce COX-2 expression and significantly block hydrogen peroxide release. The observed reduction in hydrogen peroxide production in the presence of Auraguard might be the reason behind the reduced COX-2 expression, as previously suggested [46]. Interleukin 8 (IL-8), alongside IL-1 β and other proinflammatory cytokines, are indicators of acute inflammatory events when detected in saliva [47], and their suppression will reduce inflammation and periodontitis [48]. Our study shows that both IL-8 and IL-1β are reduced in infected D6234 primary oral epithelial cells in the presence of Auraguard, suggesting that the plaque-derived bacterial pathogens will have a lesser impact on the host oral epithelium. Plant extracts are accountable for similar results on IL-8 reduction, and this pattern of reduced IL-8 secretion was demonstrated with plant extracts on HuH-7 cells [49] and Caco-2 cells [33]. It is also possible that the reduced IL-8 secretion is caused by the reduced IL-1β secretion, as it has been reported that IL-1β mediates IL-8 secretion in some instances [33]. Specifically related to oral health, in vitro herbal extracts can indeed modulate gingival cytokine expression in response to bacterial infection and reduce the host pro-inflammatory signals in gingival epithelial cells such as IL-8 and IL-1β [50].

Clearly there is a link between H_2_O_2_ and the modulation of pro-inflammatory events in eukaryotic cells, indicating that in its absence the levels of inflammatory cytokines might be reduced [51]. Well, such observations have been previously reported and directly related to the use of natural antimicrobial mixtures [35], with clear suggestions that they can block cellular signaling pathways (e.g., the ERK pathway) and mediate a reduction in pro-inflammatory responses as a result of bacterial infections [52,53]. Our study also shows that in the presence of significantly reduced H_2_O_2_ levels, caused by the antimicrobial mixture, the pathogens are unable to activate the COX-2 mediator and cause a pro-inflammatory response in the host cells. Host-released H_2_O_2_ also mediates bacterial virulence and is particularly known for its ability to modulate enzyme activity via post-translational modifications, leading to loss of bacterial EPS formation [54]. Extracellular polysaccharides (EPSs) offer increased resistance to environmental stress, are key molecules involved in biofilm formation and can increase resistance to antibiotics and to the host immune response [55]. In the case of bacteria only, the natural antimicrobial mixture was also able to disrupt the cell membrane integrity, an effect also previously described in relation to the effect of carvacrol on *S. pyogenes* [16]. The results reported herein also indicate a significant reduction in exopolysaccharide production and in H_2_O_2_ released by the *S. aureus*-, *S. pyogenes*- and *E. faecalis*-infected D6234 primary oral epithelial cells. Moreover, at a larger scale, the impact of plant extracts (gallic acid) on *Streptococcus* spp. and *Staphylococcus* spp. EPS production is significant (over 80%), as recently reported [56]. Undoubtedly, based on the existing information, we can further hypothesize that the inclusion of natural antimicrobials, such as Auraguard, can constitute an efficient solution to treat bacterial plaque biofilms and prevent infections caused by the plaque resident bacteria.

## 5. Conclusions

**Figure 6:**
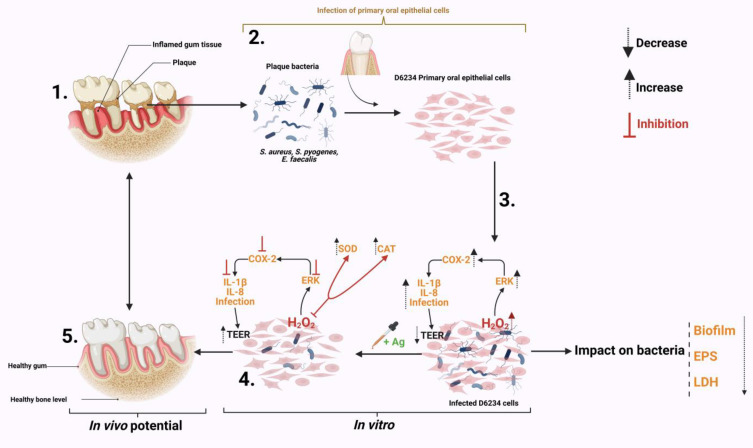
Summary of the impact Auraguard on *S. aureus*, *S. pyogenes* and *E. faecalis* in an in vitro canine oral primary epithelial cell infection model. (**1**) The appearance of dental plaque and inflamed gum tissue. (**2**) Isolated plaque bacteria (*S. aureus*, *S. pyogenes* and *E. faecalis*) and isolated D6234 primary oral epithelial cells from healthy gum tissue. (**3**) Infection of D6234 cells with *S. aureus*, *S. pyogenes* and *E. faecalis* in the presence of 0.125% Ag and the subsequent metabolic effects. (**4**) Lower infection rates following infection. (**5**) Expected in vivo intervention results. Created with Biorender.com.

The results presented in this study describe the impact of a natural antimicrobial mixture (organic acids) on the oxidative control of a pro-inflammatory response in *S. aureus*-, *S. pyogenes*- and *E. faecalis*-infected canine primary oral epithelial cells (D6234). As shown in Figure 6 the antimicrobial mixture has a triple effect during infection. First, by attenuating some of the bacterial virulence factors (EPS, biofilm) and weakening bacterial membranes. Secondly, the antimicrobial mixture can reduce the pathogen’s ability to adhere to D6234 cells and increase epithelial tight junctions without any impact on cell viability. Thirdly, the antimicrobials were able to reduce the inflammatory effect and the oxidative stress in infected cells and inhibit the oxidative pathways involved in controlling the pro-inflammatory response. The antioxidative effect is achieved by inhibiting the externally regulated kinases (ERKs), which play an important role in cell survival during oxidative stress and are involved in controlling the immune response through oxidative dephosphorylation. In regard to their future potential, we predict (as indicated in Figure 6) that, in vivo, the antimicrobials will be able to reduce gum inflammation and oxidative stress caused by plaque resident bacterial pathogens such as *S. aureus*, *S. pyogenes* and *E. faecalis*, and significantly prevent plaque formation.

## Figures and Tables

**Table 1:** Minimum inhibitory concentration (MIC) and minimum bactericidal concentration (MBC) activity of the antimicrobial mixture.

Strains	MIC (%)	MBC (%)
*S. aureus*	0.25	0.50
*S. pyogenes*	0.25	0.50
*E. faecalis*	0.25	0.50

## Data Availability

Not applicable.
